# Bacterial Substrate Transformation Tracked by Stable-Isotope-Guided NMR Metabolomics: Application in a Natural Aquatic Microbial Community

**DOI:** 10.3390/metabo7040052

**Published:** 2017-10-19

**Authors:** Mario Uchimiya, Yuuri Tsuboi, Kengo Ito, Yasuhiro Date, Jun Kikuchi

**Affiliations:** 1 RIKEN Center for Sustainable Resource Science, 1-7-22 Suehiro, Tsurumi, Yokohama, Kanagawa 230-0045, Japan; yuuri.tsuboi@riken.jp (Y.T.); kengo.ito@riken.jp (K.I.); yasuhiro.date@riken.jp (Y.D.); 2 Graduate School of Medical Life Science, Yokohama City University, 1-7-29 Suehiro, Tsurumi, Yokohama, Kanagawa 230-0045, Japan; 3 Graduate School of Bioagricultural Sciences and School of Agricultural Sciences, Nagoya University, 1 Furo, Chikusa, Nagoya 464-8601, Japan

**Keywords:** aquatic environment, heterotrophic bacteria, intracellular metabolite, metabolomics, nuclear magnetic resonance spectroscopy, stable-isotope labeling

## Abstract

The transformation of organic substrates by heterotrophic bacteria in aquatic environments constitutes one of the key processes in global material cycles. The development of procedures that would enable us to track the wide range of organic compounds transformed by aquatic bacteria would greatly improve our understanding of material cycles. In this study, we examined the applicability of nuclear magnetic resonance spectroscopy coupled with stable-isotope labeling to the investigation of metabolite transformation in a natural aquatic bacterial community. The addition of a model substrate (^13^C_6_–glucose) to a coastal seawater sample and subsequent incubation resulted in the detection of >200 peaks and the assignment of 22 metabolites from various chemical classes, including amino acids, dipeptides, organic acids, nucleosides, nucleobases, and amino alcohols, which had been identified as transformed from the ^13^C_6_–glucose. Additional experiments revealed large variability in metabolite transformation and the key compounds, showing the bacterial accumulation of glutamate over the incubation period, and that of 3-hydroxybutyrate with increasing concentrations of ^13^C_6_–glucose added. These results suggest the potential ability of our approach to track substrate transformation in aquatic bacterial communities. Further applications of this procedure may provide substantial insights into the metabolite dynamics in aquatic environments.

## 1. Introduction

In natural aquatic environments, heterotrophic bacteria transform large quantities of organic substrates inside cells, constituting one of the key processes of material cycling on a global scale [1]. Detailed information on bacterial metabolite transformation in aquatic environments would contribute greatly to our understanding of the regulatory mechanisms of global material cycles. However, the wide range of organic molecules produced by natural aquatic bacteria, which is greatly affected by the variable bacterial metabolism and local environmental conditions [1], has hindered the preselection of key metabolites and choice of appropriate analytical approaches. Therefore, the development of procedures for the comprehensive detection of metabolite transformation by natural aquatic bacteria without the need for presupposition of target metabolites is required.

The recently emerging environmental metabolomics has the potential to extract comprehensive metabolite information without the need to predict key metabolites in a system [2]. Mass spectrometry (MS) and nuclear magnetic resonance (NMR) spectroscopy are the two approaches used most widely in metabolomics, and both have been applied complementarily, extracting the advantages of each method [3,4]. Of the two approaches, NMR metabolomics requires no or less sample derivatization, preseparation, and purification processes [4], and has been applied in various fields [5,6,7], including aquatic systems (e.g., [8,9]), to extract the bulk properties of metabolites. Especially, NMR metabolomics coupled with the stable-isotope labeling of compounds (stable-isotope-guided NMR metabolomics), which enhances the detectability of NMR, has been used to elucidate the metabolite transformation patterns of various biota (e.g., [10,11,12]), including those of bacteria (e.g., [13,14]). However, most studies have focused mainly on either bacterial isolates or the gut bacterial community (broadly reviewed by Tang et al., 2012 [15]). To the best of our knowledge, there have been no studies of stable-isotope-guided NMR metabolomics that attempted to assign metabolites transformed by an aquatic bacterial community nor have studies tested whether this approach has the ability to detect key compounds in the transformation of metabolites by aquatic bacteria. Therefore, the applicability of stable-isotope-guided NMR metabolomics to address metabolite transformation in natural aquatic bacteria remains unclear. The development of stable-isotope-guided NMR metabolomics targeting natural aquatic microbial communities may provide broader insight into the metabolite dynamics in aquatic environments.

The objective of this study was to examine whether stable-isotope-guided NMR metabolomics can be used to investigate metabolite transformation in a natural aquatic bacterial community. To accomplish this, we addressed two points: (1) the assignment of metabolites transformed from a model substrate by natural aquatic bacteria (Experiment 1) and (2) the extraction of key compounds that contribute to the variability in transformed metabolites (Experiment 2). For Experiment 1, we collected a seawater sample from a coastal location, incubated the sample for 2 days after adding ^13^C_6_–glucose, and analyzed bacterial intracellular metabolites using NMR. Based on the information obtained in Experiment 1, the variability in the composition of the transformed metabolites was examined using samples incubated for different periods or with different concentrations of ^13^C_6_–glucose, and key metabolites contributing to the variability were extracted in Experiment 2. Errors in the detection of bacterial metabolite transformation patterns inherent to our experimental approach, and the potential influences of background compounds on the results, were also examined. The results of these experiments indicated that stable-isotope-guided NMR metabolomics has the potential to track metabolite transformation in an aquatic bacterial community.

## 2. Results

### 2.1. Experiment 1: Assignment of Metabolites Transformed by a Natural Aquatic Bacterial Community

**Figure 1:**
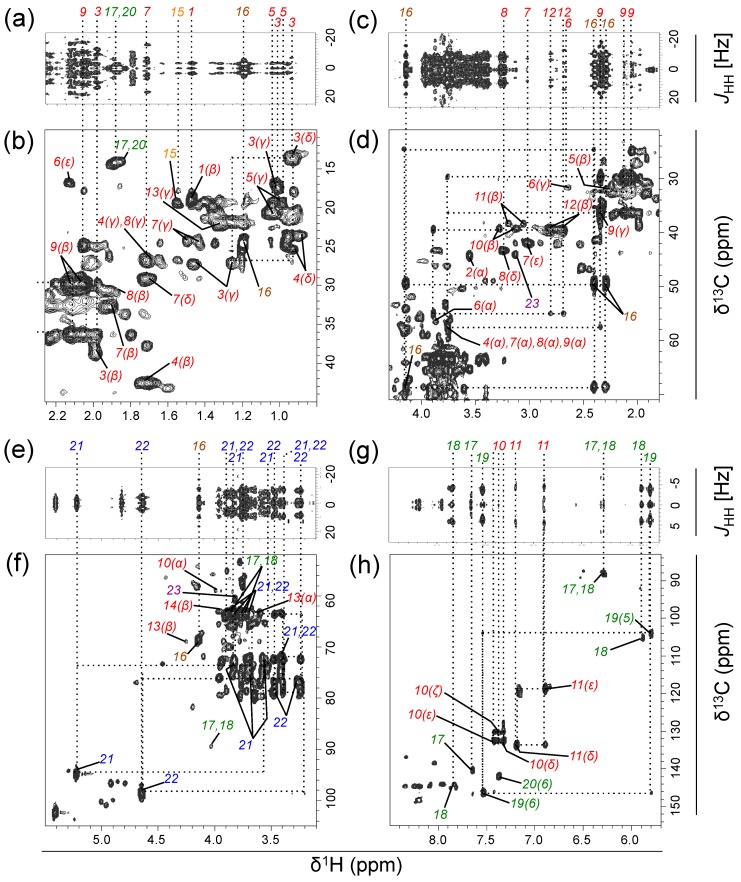
^13^C-decoupled two-dimensional ^1^H‒^1^H *J*-resolved spectroscopy (2D-*J*res-dc) (**a**,**c**,**e**,**g**) and ^1^H‒^13^C heteronuclear single quantum coherence‒total correlation spectroscopy (HSQC‒TOCSY) (**b**,**d**,**f**,**h**) spectra for metabolites transformed from ^13^C_6_–glucose by a natural aquatic bacterial community (final concentration added, 500 µM C) (Experiment 1). Signals were obtained after a 2-day incubation of a seawater sample collected from a coastal location. Spectra for the δ^1^H (δ^13^C) ranged from 0.8 to 2.26 (10–45) (**a**,**b**); 1.8–4.3 (21–71) (**c**,**d**); 3.1–5.5 (52–105) (**e**,**f**); and 5.7–8.5 (83–155) ppm (**g**,**h**) are shown. Numbers indicate the corresponding metabolites listed in Table 1 (amino acids: red; dipeptides: orange; carboxylic acids: brown; nucleosides and nucleobases: green; carbohydrates: blue; amino alcohols: purple). Greek letters or numbers in parentheses for amino acids and nucleobases, respectively, denote the atom designation. In (**a**), an increased (×6) contour intensity magnitude was used for the δ^1^H range 1.6–2.26 ppm relative to the rest of the field (0.8–1.6 ppm). For the detailed analytical settings, see Table S1.

**Figure 2:**
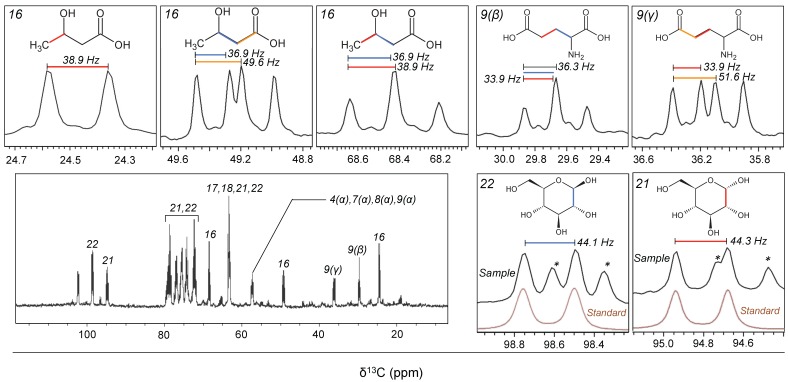
One-dimensional ^13^C-spectrum for the seawater sample incubated over 2 days after the addition of ^13^C_6_–glucose (Experiment 1). Patterns in the ^13^C–^13^C coupling (^1^*J*cc) for the major metabolites are shown as expanded panels. Italic numbers indicate the corresponding metabolites listed in Table 1. Greek letters in parentheses for amino acids denote the atom designation. For detailed analytical settings, see Table S1. In the panels showing the anomeric carbon of glucose (21 and 22), a spectrum determined for a standard compound of ^13^C_6_–glucose is also shown (in brown), because the sample spectrum overlapped unknown signals (asterisks). Chemical structures were drawn by ACD/ChemSketch (ACD/Labs).

The ^1^H‒^13^C heteronuclear single quantum coherence (HSQC) spectrum contained 222 peaks and SpinAssign identified 70 candidate metabolites (tolerance ±0.03 and ±0.53 ppm for the ^1^H and ^13^C dimensions, respectively; SpinAssign *p*-value, >1 × 10^−^^20^). Of the annotated metabolites, 22 compounds (77 peaks) were further assigned based on the correlation signals determined by ^1^H‒^13^C HSQC‒total correlation spectroscopy (HSQC‒TOCSY) and spin–spin coupling constants (*J*-coupling values) determined by ^13^C-decoupled two-dimensional ^1^H‒^1^H *J*-resolved spectroscopy (2D-*J*res-dc) pulse programs (Figure 1 and Table 1). These assigned metabolites belonged to various chemical classes, including proteinogenic amino acids, dipeptides, carboxylic acids, nucleosides, nucleobases, carbohydrates (including anomers), and amino alcohols. To examine the detailed coupling patterns of the detected metabolites, one-dimensional ^13^C-spectrum measurement (1D–^13^C) was conducted. The 1D–^13^C showed splitting of each peak derived from the ^13^C−^13^C coupling (^1^*Jcc*), and signals derived from non-labeled compounds were uncommon (Figure 2), indicating that the detected metabolites were largely ^13^C-labeled. The low signal of background compounds was confirmed by incubating the original seawater without adding ^13^C_6_–glucose, and the signal intensities were significantly lower than those incubated after ^13^C_6_–glucose addition (Figure S1). The variability in the signals among replicate samples (*n* = 3) was on average 9.7% for major metabolites (standard deviation ± 4.3) (Figure S1).

### 2.2. Experiment 2: Variability in the Composition of Metabolites Transformed by Natural Aquatic Bacteria

**Figure 3:**
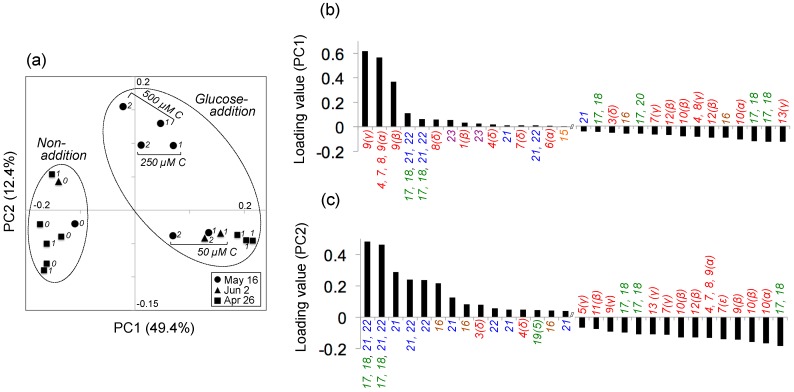
(**a**) Results of principal component analysis of the variability in metabolite composition of samples incubated for different periods and with different ^13^C_6_–glucose concentrations. Metabolites were transformed from ^13^C_6_–glucose by a natural aquatic bacterial community, using coastal seawater samples collected on 16 May 2016, 2 June 2016, and 26 April 2017. Numbers in italics beside the symbols indicate the incubation period (days). Non-addition: treatment in which the original seawater was incubated without added ^13^C_6_–glucose; glucose-addition: treatment in which the seawater sample was incubated after adding ^13^C_6_–glucose (50, 250, or 500 µM C). Loading values for principal components (**b**) 1 (PC1) and (**c**) 2 (PC2), showing the top and bottom 15 metabolite signals. Numbers indicate the corresponding metabolites listed in Table 1 (amino acids: red; dipeptides: orange; carboxylic acids: brown; nucleosides and nucleobases: green; carbohydrates: blue; amino alcohols: purple). Greek letters or numbers in parentheses for amino acids and nucleobases, respectively, denote the atom designation. See also Figure S2 for the compounds and chemical classes shown in (**b**,**c**). For detailed analytical settings, see Table S1.

Principal component analysis (PCA) indicated that 62% of the variability in the metabolite peak composition was explained by the combination of principal components 1 (PC1) and 2 (PC2) (49.4 and 12.4%, respectively) (Figure 3a). The experiment examining the variability (i.e., standard deviation) in metabolite composition among triplicate samples (26 April) indicated that the replicate variability was relatively low (average standard deviation among replicates, 0.01 ± 0.01 and 0.03 ± 0.03 for PC1 and PC2 loading values, respectively) compared with those for all samples (range: −0.2–0.2 and −0.1–0.2 for corresponding values, respectively; Figure 3a). The composition of bacterial metabolites after adding ^13^C_6_–glucose and the subsequent 1–2-day incubation was characterized by increased PC1 values relative to those in the initial samples and samples incubated without added ^13^C_6_–glucose (Figure 3a). Based on the metabolite assignments in Experiment 1 and the PC1 loading value output for each metabolite peak, this distribution was most influenced by the increased signals related to glutamate (Figure 3b; Figure S2). The experiments examining the effect of different substrate concentrations on the bacterial metabolite composition (16 May) also exhibited large variability in the composition of bacterial metabolites, in which the metabolite compositions of samples treated with 250 and 500 µM C ^13^C_6_–glucose were characterized by greater PC2 values relative to those treated with 50 µM C ^13^C_6_–glucose (Figure 3a), for which the distribution was markedly affected by increased signals related to carbohydrate (glucose) and carboxylic acid (3-hydroxybutyrate) (Figure 3a,c; Figure S2).

## 3. Discussion

This study first examined the applicability of stable-isotope-guided NMR metabolomics to track metabolite transformation in aquatic bacterial communities. The metabolites assigned in this study are consistent with those reportedly produced by heterotrophic bacteria [16]. The ^13^C–^13^C coupling patterns of metabolite peaks that were evident after the incubation indicated that the added ^13^C_6_–glucose was incorporated into bacterial cells and various metabolites originated from the ^13^C_6_–glucose. The coupling patterns also indicated that the detected metabolites were strongly ^13^C-labeled and the signals generally reflected the amount of metabolites transformed by the bacteria. The wide range of compounds assigned in the experiment suggests that carbon initially supplied as ^13^C_6_–glucose entered the central pathways (glycolysis pathway and tricarboxylic acid cycle) to generate amino acids, organic acids, and nucleobases [16]. Based on the results of Experiment 2, the composition of these metabolites can vary markedly with the incubation period and the concentration of ^13^C_6_–glucose added to the samples. When ^13^C_6_–glucose was added, the main compound that accumulated in cells with increasing incubation time was glutamate. Glutamate production is the primary gate of ammonium assimilation in marine bacteria [17]. This assimilation is catalyzed by glutamine synthetase and glutamate synthase (GS/GOGAT pathway) and by glutamate dehydrogenase (GDH pathway) [17]. Glutamate is the major end product of these pathways and is used to produce other nitrogen-containing compounds. In this study, we used a non-nitrogen-containing compound (i.e., glucose) as a substrate for bacteria. Therefore, the dominant glutamate production might be a reflection of the active uptake of ammonium to compensate for the deficiency in nitrogen. Notably, enhanced ammonium uptake with the addition of glucose has been demonstrated in a marine bacterial community [18]. The experiment examining the effect of increasing the concentration of ^13^C_6_–glucose resulted in a greater intracellular accumulation of glucose and 3-hydroxybutyrate, instead of glutamate. 3-Hydroxybutyrate is one of the constituents of polyester poly-β-hydroxyalkanoate, which functions as a carbon and energy reservoir in bacteria [19]. 3-Hydroxybutyrate can be produced from glucose after glycolysis, and the addition of glucose was reported to result in the production of 3-hydroxybutyrate in marine bacteria (e.g., [20]). Importantly, the bacterial production of 3-hydroxybutyrate is known to be stimulated under nutrient limitation [21]. Therefore, one possible explanation for the enhanced production of 3-hydroxybutyrate instead of glutamate might be the induction of nitrogen depletion due to the excess addition of glucose. Although we did not have any data on nutrients, these two inferences regarding metabolite production related to nutrient availability require further testing in future studies. Overall, the variability in metabolite transformation patterns, and the key metabolites of aquatic bacterial communities could be detected by our experimental procedures and analytical settings.

Potential concerns regarding the interpretation of the metabolite transformation patterns derived using our approach, and related recommendations, are as follows. (1) The detection of bacterial metabolites by NMR is highly solvent-dependent [22], so the solvent should be chosen carefully depending on the study focus. We used a polar solvent (D_2_O) to address the transformation of a hydrophilic compound (glucose), which might in turn result in less sensitive detection of aliphatic compounds [22]. Parallel measurements of bacterial metabolites using a semipolar organic solvent would provide additional information to obtain the bulk properties of the bacterial metabolite transformation processes. (2) Because we used filters when collecting bacterial cells, metabolites that bacteria tend to excrete or secrete extracellularly were missing. Procedures should be introduced to collect the dissolved fraction of organic compounds from aquatic samples [23,24,25] to detect both intra- and extracellular metabolites of interest. (3) Before the experiment, we pre-filtered the sample through 1.0 µm filters to remove large microbes, intending to avoid the occurrence of their metabolite transformation in our samples. However, this could potentially lead to the selection of the microbial community that participated in the community metabolism of the system. Parallel examination of the extent of changes in community structure due to the filtration step would help to evaluate the representativeness of this result. (4) This study assigned bacterial metabolites based on a combination of chemical shifts, ^1^H‒^13^C correlation signals, and *J*-coupling values. In addition, peaks overlapping with unknown signals were separated based on parallel measurements of a standard compound (Figure 2). However, to increase the accuracy of the assignment, it would be beneficial to conduct spiking experiments and validate the results. (5) In this study, 65% of the detected peaks remained unassigned. This might be improved as the database is updated [4] and by introducing additional multidimensional NMR approaches [6].

Although these potential limitations inherent to our experimental approach should be considered, to our knowledge, this is the first study to assign metabolites transformed by aquatic bacterial communities and examine the variability, based on stable-isotope-guided metabolomics. Due to the superior comparability of the data collected by NMR metabolomics [26,27], the information regarding peak assignment provided here would be informative for future studies directly comparing the metabolite properties across various fields or laboratories. Future possible directions expanding our ability to extract metabolite transformation in aquatic microbial communities are as follows. First, our method could be used complementarily with the alternative approach of MS-based metabolomics [3,4]. Each metabolomics outputs a unique metabolite list [28], and linking of the two approaches would substantially improve our coverage of metabolite transformation processes. Second, although the detected metabolites were strongly ^13^C-labeled in this study, our approach could be applied to examine detailed metabolic pathways by adjusting the metabolite labeling conditions (e.g., [12]). For example, a recent study based on bacterial isolates suggested that the Entner–Doudoroff (ED) pathway, rather than the Embden–Meyerhof–Parnas (EMP) pathway, is dominant in marine bacteria in transforming glucose [29], which contrasts terrestrial bacteria. Testing whether this process is generally dominant in marine bacterial communities could be done by examining coupling patterns after the appropriate adjustment of the concentrations or labeling ratios of the substrates to be used in the experiment. Third, given the large variability in metabolite transformation patterns detected in this study, it would be worthwhile to integrate metabolite information with bacterial parameters (e.g., cell density; physiological condition; community structure). Especially, it would be worthwhile to take advantage of stable-isotope labeling, coupling our approach with deoxyribonucleic acid stable-isotope probing (DNA-SIP) [30], which has been applied in a gut bacterial community [31], but has yet to be conducted in an aquatic bacterial community. This may clarify the taxa actively incorporating the ^13^C-labeled substrate, and those that tend to use downward transformed products. In summary, this study demonstrated that stable-isotope-guided NMR metabolomics provides valuable information on metabolite transformation in the aquatic bacterial community. Further applications of this procedure and coupling with alternative approaches may provide substantial insights into bacterial metabolite dynamics in aquatic environments.

## 4. Materials and Methods

### 4.1. Sample Collection, Incubation, and Processing

Seawater samples used for the incubation experiments were collected from a coastal location (35.48° N, 139.69° E) on 16 May 2016 (Experiments 1 and 2), 2 June 2016 (Experiment 2), and 26 April 2017 (Experiment 2). In the laboratory, the collected samples were filtered through a 1.0 µm Omnipore filter (Millipore) to remove large microbes, and the filtrate was transferred to clean 1 L polypropylene bottles. [*U*-99% ^13^C_6_]-d–Glucose (CLM-1396, Cambridge Isotope Laboratories, Tewksbury, MA, USA) was added to the sample [final concentration, 50–500 µM C (8–83 µM ^13^C_6_–glucose), depending on the experiment], and the sample was incubated in the dark at in situ temperature for 2 days. Subsamples were collected from the bottles after incubation for 0, 1, or 2 days, and 390–1000 mL of the sample was filtered, and the bacterial cells were concentrated using a 0.2 µm Omnipore filter (Millipore). Before filtration, each filter was washed with 300 mL of Milli-Q water (Millipore) to reduce blank signals derived from the product. Then, the filter sample was soaked with 15 mL of Milli-Q water in a 50 mL tube (Falcon), and the bacterial cells were disrupted using an ultrasonicator (Bioruptor, Diagenode) on ice. This process was repeated three times, and the sample was freeze-dried after removing the filter from the tube. The dried sample was dissolved in phosphate buffer (pH 7.0; 60 mmol L^−1^ K_2_HPO_4_ and 40 mmol L^−1^ KH_2_PO_4_ dissolved in D_2_O, WAKO), which was amended using a calibration standard of 1 mmol L^−1^ 2,2-dimethyl-2-silapentane-5-sulfonate (DSS). During sample collection and processing, gloves were worn, and care was taken not to contaminate the samples.

### 4.2. Metabolite Detection Using NMR

Bacterially transformed metabolites were determined using the Bruker AVANCE II DRU 700 NMR spectroscopy equipped with a cryogenic probe operating at 700.15 and 176.06 MHz for ^1^H and ^13^C, respectively. Measurements were made at 298 K using pulse programs for ^1^H‒^13^C HSQC (formally named hsqcetgpsisp2.2 by Bruker), ^1^H‒^13^C HSQC‒TOCSY (hsqcdietgpsisp.2), ^1^H‒^1^H 2D-*J*res (jresgpprqf), 1D–^13^C (zgig), and modified 2D-*J*res, which contains a ^13^C decoupling pulse (2D-*J*res-dc) (see Table S1 for detailed analytical settings). The data were processed using TopSpin 3.5 software (Bruker). In Experiment 1, the peaks from the HSQC spectrum were searched for in the SpinAssign database (http://dmar.riken.jp/spinassign/) [32]. The HMDB ([33], http://www.hmdb.ca/) and BMRB ([34], http://www.bmrb.wisc.edu/metabolomics/) databases were used as supporting information. Metabolites were further assigned by integrating the HSQC‒TOCSY correlation signal data and the chemical shift (δ^1^H) and *J*-coupling value determined by 2D-*J*res-dc. For the δ^1^H and *J*-value of each metabolite, the SpinCouple ([35], http://dmar.riken.jp/spincpl/), HMDB, and BMRB databases were used. In Experiment 2, the HSQC data collected in the experiments conducted on 16 May 2016, 2 June 2016, and 26 April 2017 were used. To examine the variability in the metabolite signal composition among samples, the peak intensity was obtained using rNMR [36], and PCA was performed using R software (version 3.3.0) [37]. In processing the results, a blank value (*n* = 3), which was derived from the filter product and determined by filtering Milli-Q water instead of sample, was subtracted from the detected value of the sample.

## Figures and Tables

**Table 1:** Summary of the metabolites assigned in this study using a combination of multiple pulse programs (see text for details). Metabolites were products of ^13^C_6_–glucose transformed by a natural aquatic bacterial community during a 2-day incubation of a seawater sample collected from a coastal location (Experiment 1). Colors: amino acids: red; dipeptides: orange; carboxylic acids: brown; nucleosides and nucleobases: green; carbohydrates: blue; amino alcohols: purple.

Chemical Class	Compound	Reference Number ^1^
Amino acids	Alanine	1
	Glycine	2
	Isoleucine	3
	Leucine	4
	Valine	5
	Methionine	6
	Lysine	7
	Arginine	8
	Glutamate	9
	Phenylalanine	10
	Tyrosine	11
	Aspartate	12
	Threonine	13
	Serine	14
Dipeptides	Alanylalanine	15
Carboxylic acids	3-Hydroxybutyrate	16
Nucleosides	Thymidine	17
	Deoxyuridine	18
Nucleobases	Uracil	19
	Thymine	20
Carbohydrates	Glucose	21(α), 22(β)
Amino alcohols	Ethanolamine	23

^1^ In Figure 1, Figure 2 and Figure 3 and Figure S1.

## Supplementary Materials

The following are available online at www.mdpi.com/2218-1989/7/4/52/s1. Figure S1: ^1^H‒^13^C HSQC analysis examining the background signals and variability among replicate samples; Figure S2: Compounds and chemical classes for Figure 3; Table S1: Summary of the analytical settings used for the NMR measurements.
