# Supplementary material for: Bacterial Substrate Transformation Tracked by Stable-Isotope-Guided NMR Metabolomics: Application in a Natural Aquatic Microbial Community
Source: Metabolites. 2017 Oct 19;7(4):52. doi: 10.3390/metabo7040052 (PMC5746732; doi:10.3390/metabo7040052)
Supplement: Supplementary file 1 [file metabolites-07-00052-s001.pdf]

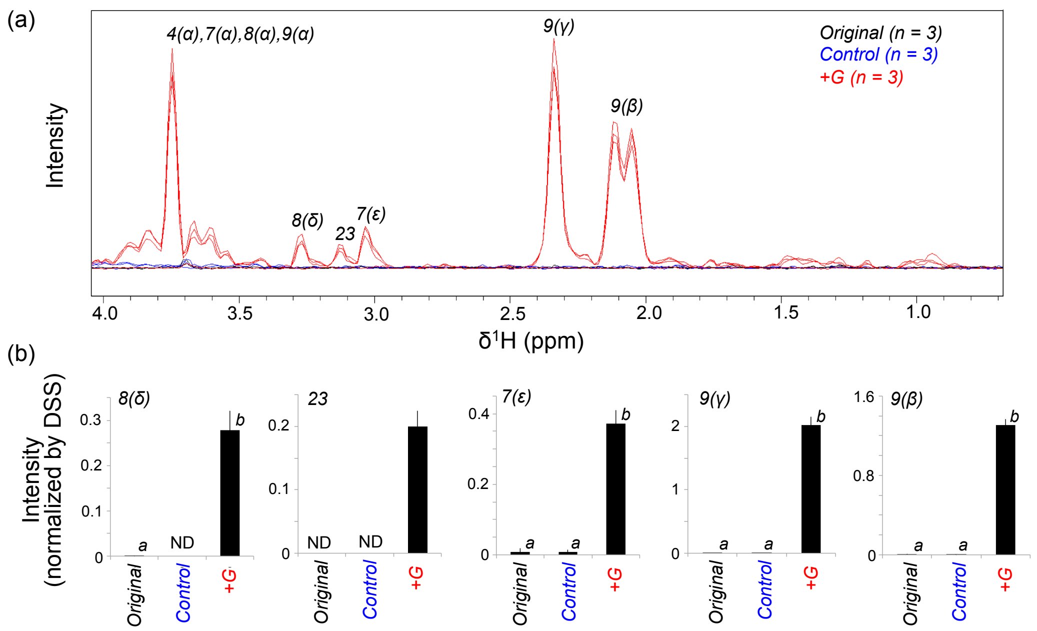

Figure S1. (**a**) Projection (f2 dimension) of the ^{1}H–^{13}C heteronuclear single quantum coherence (HSQC) spectrum for the experiment examining the background signals derived from original seawater, and the variability among replicate samples. The seawater sample was collected from a coastal location on 26 April 2017, and the collected sample was divided into three bottles (i.e., in triplicate) for each treatment. Three treatments were prepared (i.e., nine bottles in total): Original: original seawater; Control: control treatment in which the original seawater was incubated for 1 day without the addition of $^{13}C_6$-glucose; +G: $^{13}C_6$-glucose-addition treatment in which the original seawater was incubated after the addition of $^{13}C_6$-glucose (final concentration: 500 μM C). After the incubation, each bottle-contained sample was filtered, and bacterial metabolites were extracted independently according to the procedure described in Section 4.1. Italic numbers indicate the corresponding metabolites listed in Table 1. Greek letters in parentheses for amino acids denote the atom designation. For the detailed analytical settings, see Table S1. (**b**) The intensity of major metabolite peaks was determined by ^{1}H–^{13}C HSQC for each treatment, and normalized by the intensity of calibration standard (DSS) to compare the metabolite signals among samples. The error bar represents the standard deviation for replicate samples (n = 3). Different italic lower case letters denote a significant difference between the treatments (either t-test or multiple comparison with Bonferroni correction, $p < 0.001$). ND: not detected.

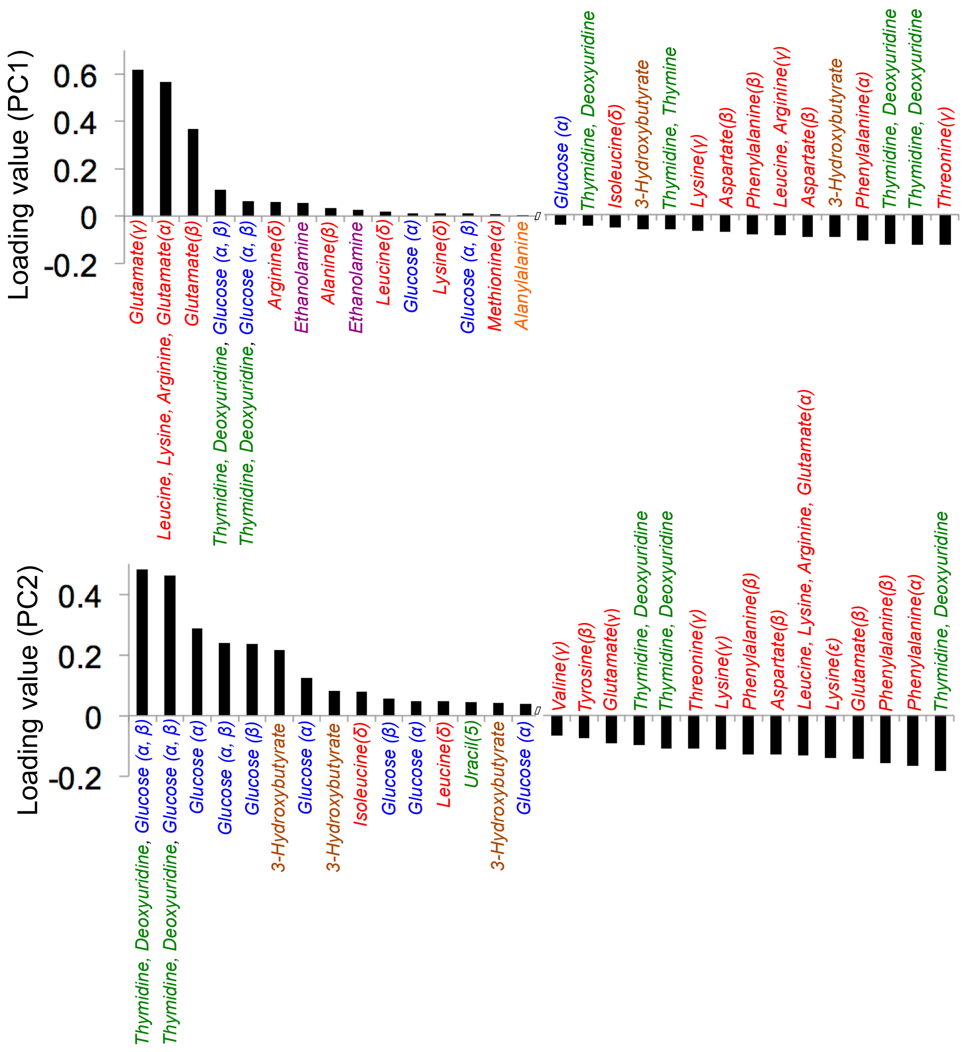

Figure S2. Compounds and chemical classes for Figures 3b,c. For detailed information, see the legend for Figure 3.

Table S1. Summary of the analytical settings used for the NMR measurements. 2D-*J*res-dc: ^{13}C-decoupled two-dimensional ^{1}H–^{1}H *J*-resolved spectroscopy; ^{1}H–^{13}C HSQC–TOCSY: ^{1}H–^{13}C heteronuclear single quantum coherence–total correlation spectroscopy; 1D–^{13}C: one-dimensional ^{13}C.

Figure	Pulse Program	Spectral Width (ppm)	Offset (ppm)	Number of Sampling Points	Number of Scans
1a,c,e,g	2D-*J*res-dc	0.07 (f1), 17 (f2)	4.7 (f2)	32 (f1), 2048 (f2)	128
1b,d,f,h	^{1}H–^{13}C HSQC–TOCSY	190 (f1), 14 (f2)	90 (f1), 4.7 (f2)	256 (f1), 1024 (f2)	128
2	1D–^{13}C	242	100	65536	512
3, S1	^{1}H–^{13}C HSQC	40 (f1), 14 (f2)	68 (f1), 4.7 (f2)	96 (f1), 1024 (f2)	160
